# Long-term and acute safety of a novel orally administered combination drug product containing milbemycin oxime and lotilaner (Credelio^®^ Plus) in juvenile and adult dogs

**DOI:** 10.1186/s13071-021-04760-z

**Published:** 2021-05-28

**Authors:** Kari L. Riggs, Scott Wiseman

**Affiliations:** Elanco Animal Health, 2500 Innovation Way, Greenfield, IN 46140 USA

**Keywords:** Credelio^®^ Plus, Lotilaner, Milbemycin oxime, Safety, Canine

## Abstract

**Background:**

The combination of milbemycin oxime (MO) and lotilaner (Credelio^®^ Plus) is a novel systemic endectocide that provides month-long effectiveness in dogs after a single oral treatment. The safety of Credelio^®^ Plus flavored chewable tablets was investigated in three target animal safety studies. Two studies (one in juveniles and one in adults) evaluated the long-term safety, and one study evaluated the acute safety of the product when administered orally at the upper end of the recommended dose range (0.75–1.53 mg/kg MO and 20–41 mg/kg lotilaner) and multiples of this dose.

**Methods:**

The objectives of these studies were to determine the long-term and acute safety of MO and lotilaner flavored chewable tablets in healthy dogs. All three studies were randomized, blinded, parallel-group design studies in healthy Beagle dogs. In each of the two long-term studies, 32 dogs were randomized among four groups to untreated controls or to treated groups at target doses of 1X, 3X, or 5X. Treatment was administered on seven (adult dogs) or nine (juvenile dogs) occasions with dosing every 4 weeks. In the acute study, 48 dogs were randomized among four groups to untreated controls or to treated groups at 1X, 3X, or 6X. In all three studies, the control group was administered placebo tablets. All dogs were fed 30 to 45 min prior to treatment and the assessment of safety was based on health observations, complete physical/neurological examinations, and food consumption. For the long-term safety studies, safety assessments also included clinical pathology evaluations (hematology, clinical chemistry and urinalysis), body weight, pharmacokinetic blood collections, and macroscopic and microscopic examinations of collected tissues.

**Results:**

MO and lotilaner did not induce any treatment-related adverse effects based on health observations, physical/neurological examinations, or food consumption in the long-term or acute studies. Additionally, in the long-term studies, MO and lotilaner did not induce any treatment-related effects on clinical pathology, body weight, and macroscopic and microscopic examinations.

**Conclusions:**

These three studies demonstrate that Credelio^®^ Plus has a wide safety margin when administered at monthly intervals to puppies and dogs at the high end of the commercial dose band.

**Graphic Abstract:**

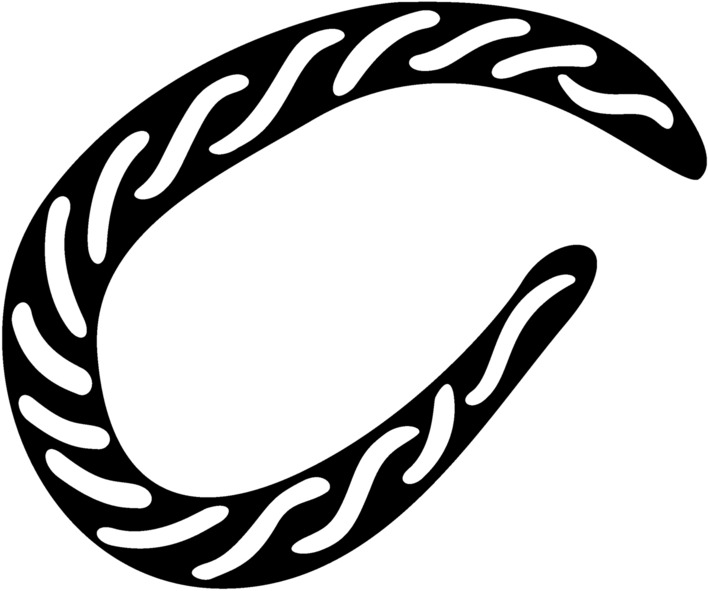

## Background

Lotilaner is an ectoparasiticide and is one of several similar drug products contained in this newer chemistry class called the isoxazolines. Lotilaner was previously developed as an oral monthly administered chewable tablet for use in dogs and cats as a mono-use drug product (Credelio^®^, Elanco Animal Health). Credelio^®^ has been shown to provide fast and consistent month-long effectiveness against fleas and ticks in dogs and cats [1–3]. It was also shown to have effectiveness against *Demodex* spp. mites in dogs [4].

Milbemycin oxime (MO) is a macrocyclic lactone (ML) that was originally developed to treat adult intestinal nematode infections in dogs (*Trichuris vulpis, Toxascaris leonina*, *Ancylostoma caninum* and *Toxocara canis*) and also used for monthly heartworm (*Dirofilaria immitis*) prevention when dosed orally at a minimum effective dose of 0.5 mg/kg [5, 6]. It was subsequently shown to be effective in killing larval and immature adult stages of *Ancylostoma caninum* and *Toxocara canis* at a minimum oral dose of 0.75 mg/kg [7]. Heartworm (*Dirofilaria immitis*), a filarial parasite in dogs and other animals, is transmitted by mosquitoes and is known to cause severe and life-threatening cardiopulmonary disease [8, 9]. Historically, heartworm disease has been prevented in dogs by prophylactic treatment with several approved oral, topical, or injectable macrocytic lactone (ML) drug products [9, 10]. Additionally, it has been shown that MO will prevent angiostrogylosis by reducing the infection level of immature adult [L5] and adult stages of the French heartworm, *Angiostrongylus vasorum* [11].

With a desire to provide a novel oral chewable tablet combination formulation that includes MO and lotilaner, this new product (Credelio^®^ Plus) was developed and assessed for prevention of heartworm and lungworm disease, to control flea and tick infestations for 1 month, and to treat and control intestinal roundworms, hookworms and whipworms that are common in dogs, thus providing a significant clinical health benefit for dog owners and their pets. Tablets of this combination drug product contain a minimum dose of 0.75 mg/kg (range 0.75–1.53 mg/kg) of MO and a minimum dose of 20 mg/kg (range 20–41 mg/kg) of lotilaner. The safety studies described in this manuscript tested the “nominal” maximum dose of 1.5 mg/kg MO and 40 mg/kg lotilaner. The minor differences between the nominal maximum use dose and the actual maximum use dose would have no impact on the safety of the product as it was demonstrated to be safe at 3, 5 and 6X the nominal maximum dose.

In regions participating in the Veterinary International Cooperation on Harmonization (VICH), the assessment of the safety of a compound in the target species is a prerequisite for the registration of veterinary products according to GL 43 [12]. For this program, it was important to determine the safety of Credelio^®^ Plus in three unique situations prior to approval of the product for administration to the dog population at large. First, as juveniles have rapidly changing physiology, it is important to ensure the product does not impact their growth and that the product is safe when the drug reaches steady state concentrations (this is especially important for drugs like lotilaner with a long elimination half-life). Second, as adult dogs have higher systemic exposure to these drug substances, it was important to demonstrate the safety of the combination drug product at steady state in this population as well. Finally, understanding the safety of the product in case of accidental consumption of an entire six-pack of tablets, provides valuable information to regulatory agencies, veterinarians, and dog owners. Therefore, three target animal safety studies were conducted: (1) long-term safety evaluation in juvenile dogs, starting at 8 weeks of age (hereafter referred to as juvenile study); (2) long-term safety evaluation in adult dogs, starting at 11 months of age (hereafter referred to as adult study); and (3) acute safety evaluation in adult dogs, starting at approximately 1 year of age (hereafter referred to as acute study). In the long-term adult and juvenile safety studies, the test articles were administered at target doses of 1X, 3X, and 5X the upper limit of the recommended dose according to VICH GL 43 [12]. The 1X dose was dosed for 1, 3, or 5 consecutive days during each of the treatment intervals to maximize systemic exposure. In the acute study, the test articles were administered at 1X, 3X, and 6X the upper limit of the recommended dose as a single bolus dose. In all studies, there was a negative control group (0X), i.e., dosed with placebo tablets.

## Methods

### Type of studies

All three studies were non-clinical laboratory, randomized, controlled, blinded studies conducted at Charles River Laboratories Ashland, LLC (CRL-Ashland; Ashland, OH, USA). These studies were designed and conducted per (a) Guidance for Industry 185 Target Animal Safety for Veterinary Pharmaceutical Products VICH GL43 [12] and OECD Principles of Good Laboratory Practice [13]; (b) applicable regulations of the U.S. Food and Drug Administration (FDA) Good Laboratory Practice (GLP) Standards, 21 CFR Part 58 (October 5, 1987) [14]; and (c) study protocol and Charles River Standard Operating Procedures (SOPs). In addition, for the adult and juvenile long-term studies, bioanalytical analyses were conducted at WuXi AppTec Co., Ltd. (Shanghai, China), statistical analyses were conducted at BioSTAT Consultants, Inc. (Portage, MI, USA), and C-reactive protein analyses were conducted at AniLytics, Inc. (Gaithersburg, MD, USA), according to each test site's SOPs. All three studies were reviewed and approved by CRL-Ashland’s Institutional Animal Care and Use Committee. This manuscript was prepared in compliance with the ARRIVE Guidelines Checklist for animal in vivo experiments [15].

### Study design

In the adult and juvenile studies, following random assignment of the Beagle dogs to treatment groups, MO and lotilaner were co-administered using fixed combination tablets at target doses of 0, 1X, 3X, and 5X the upper limit of the therapeutic dose range by administering the 1X dose for 1, 3 or 5 consecutive days, respectively, every 28 days for seven (adult) or nine (juvenile) consecutive dose cycles. Necropsy occurred on day 197 or 198 (half of the dogs on each day) for the adult study and on day 256 for the juvenile study. Adult dogs were approximately 11 months of age and the juveniles were approximately 8 weeks of age at study initiation.

In the acute study, MO and lotilaner were co-administered at target doses of 0, 1X, 3X, and 6X the upper limit of the therapeutic dose range to adult Beagle dogs (at approximately 1 year of age) as a single bolus dose with dogs returned to colony at the study conclusion 48 h after dosing.

For all three studies, the route of administration was oral (by tablet) to dogs in the fed state.

## Experimental animals

### Test system

Beagle dogs were the test system as dogs are the target species and Beagles are a breed for which significant historical control data are available and recognized as appropriate for safety testing.

For each study, appropriate-age Beagle dogs were received in good health from a USDA licensed dog vendor, Marshall BioResources, North Rose, NY, USA. Each dog was examined by a veterinarian upon receipt and weighed on the following day. All dogs were appropriately immunized either by the supplier or during their time at CRL-Ashland.

Dogs considered suitable for the study were housed for a 13-day acclimation for the adult and juvenile studies and for a 14-day acclimation for the acute study. During acclimation, each dog was observed twice daily for mortality and changes in general appearance or behavior, underwent a physical examination, had clinical pathology samples collected and fecal samples collected and checked for parasites.

For the adult and juvenile studies, there were eight dogs per treatment group (four males and four females). For the acute study, there were 12 dogs per treatment group (six males and six females). At study initiation, dog weight ranges were as follows: in the adult study, between 7.7 to 10.7 kg (males) and 5.7 to 7.7 kg (females); in the juvenile study, between 2.0 to 3.4 kg (males) and 1.8 to 2.9 kg (females); and in the acute study, 7.9 to 11.8 kg (males) and 5.9 to 8.8 kg (females).

## Randomization

A few days prior to initiation of dose administration, dogs underwent a pre-study physical examination. Normal, healthy dogs were selected on the basis of physical findings (weight, body condition, health observations, and physical examination findings), clinical pathology findings, behaviors that could interfere with performance of required procedures, and acclimation data (acceptable pre-study general heath and observations, adequate feed consumption, etc.). The healthy dogs judged to be suitable for testing from each gender (16/gender for the adult and juvenile studies and 24/gender for the acute study) were blocked by weight and randomly assigned to treatment groups and cages according to a randomization plan provided by the statistical principal investigator. Each block contained four dogs of the same sex and each treatment group occurred once within each block. Within each block, the treatments were in a random order. The plan also used a completely randomized assignment of dogs to cages. Remaining dogs not assigned to the studies were transferred to the CRL-Ashland colony. For the adult and juvenile studies, in order to minimize the potential for bias at the scheduled necropsy, the order of necropsy was randomly determined, according to a list provided by the statistical principal investigator, so that equal numbers of dogs from each gender and treatment group were evaluated each day.

## Blinding

The study director and any personnel administering treatment and/or collecting clinical observations (veterinary physical observations, clinical observations, and unscheduled observations), body and food weights, or gross macroscopic examinations were blinded to dose group assignments. Blinding was maintained until conclusion of the microscopic examination of collected tissues and after assignment of VeDDRA (Veterinary Dictionary for Drug Regulatory Activities) codes by the study director. Study personnel requiring access to study group mappings were not blinded to the treatment group allocations; these included the statistical principal investigator, bioanalytical principal investigator, formulations group manager, personnel dispensing tablets, personnel processing pharmacokinetics blood samples, and quality assurance.

## Treatment

### Test article administration

The test article was a fixed combination tablet of MO and lotilaner. For the adult and juvenile studies, there were four sizes, containing 2.11/56.25, 4.22/112.5, 8.44/225, and 16.88/450 mg of MO/lotilaner per active tablet. For the acute study, there was an additional tablet containing 33.75/900 mg of MO/lotilaner per tablet. Placebo tablets were manufactured to mimic the active tablets.

For the adult and juvenile studies, to achieve the clinical multipliers while maximizing exposure and maintaining blinding, the 1X dose was administered on 1, 3, and 5 consecutive days of each dose cycle for the 1X, 3X, and 5X groups, respectively (see Table 1). On days active tablets were not administered, placebo tablets were administered to maintain blinding. A corresponding control group was administered placebo tablets for all 5 days of each dosing cycle. The first day of dosing was study day 0; the first week of dosing was study week 0.

**Table 1:** Dosing scheme utilized in the adult and juvenile target animal safety studies

Dose level	Dose administration study days				
	0, 28, 56, 84, 112, 140, 168, 196^*^, 224^*^	1, 29, 57, 85, 113, 141, 169, 197^*^, 225^*^	2, 30, 58, 86, 114, 142, 170, 198^*^, 226^*^	3, 31, 59, 87, 115, 143, 171, 199^*^, 227^*^	4, 32, 60, 88, 116, 144, 172, 200^*^, 228^*^
0X	Placebo	Placebo	Placebo	Placebo	Placebo
1X	Placebo	Placebo	Placebo	Placebo	1X
3X	Placebo	Placebo	1X	1X	1X
5X	1X	1X	1X	1X	1X

^*^ Juvenile study only

In the acute study, active or placebo tablets were administered once (bolus dosing) at 0, 1X, 3X, and 6X on study day 0.

For all three studies, dogs were provided canned food approximately 30 to 45 min prior to dosing as food has been shown to increase both lotilaner and milbemycin oxime absorption [16, 17]. Single tablets or multiple tablets were then administered to achieve as close as possible to the individual target dose based on the most recent body weights collected prior to the dosing cycle. After dosing, a small amount of water was given, and the inside of the mouth was inspected to ensure that the tablet(s) were swallowed. Dogs that vomited within 2 h of dosing were re-dosed (only 1 re-dose per day) with either fresh tablets or expelled tablets if they were able to be recovered and handled easily.

## Food and water

For the adult and acute study, dry food (Lab Diet^®^ Certified Canine Diet #5007, PMI Nutrition International, Inc, Richmond, IN, USA.) was available for at least 4 h each day during acclimation and on non-dosing days, except for a few fasting periods. Fasting occurred prior to blood collection for each scheduled clinical pathology evaluation, prior to dosing, and prior to the scheduled necropsy (adult and juvenile studies only). On dosing days, dogs were offered Alpo^®^ (commercially available canned food) at a rate equal to approximately 25% of the manufacturer’s recommended daily amount based on its body weight. If after 20 min a dog had not eaten the offered food, the dog was fed by placing small amounts of food into the back of the mouth and allowing it to swallow until the entire portion had been consumed. After dosing, dogs were offered their normal daily ration of dry food. The dogs were acclimated to the canned food and feeding procedure for several days prior to the initial dose (adult and acute study) and prior to each dosing cycle (adult study).

For the juvenile study, a similar process as described above for the adult study was followed with a few exceptions. The juvenile dry food was Lab Diet^®^ Certified Canine Diet #5L66 High Density Canine (PMI Nutrition International, Inc., Richmond, IN, USA) instead of #5007. In addition, the juveniles were weaned from the supplier diet (Eukanuba Premium Performance, Leipsic, OH, USA) to the certified canine diet during acclimation. Finally, the juvenile diet was divided into two approximately equal portions offered twice (approximately 6–8 h apart) so that they had access to food for at least 12 h each day.

For all three studies, drinking water was available ad libitum.

## Housing/environmental conditions

In the juvenile study, dogs were housed in pairs during acclimation and during the first dosing cycle (except during the 5 days of dosing where they were individually housed). In the remainder of the juvenile study and for the adult and acute studies, the dogs were housed individually in clean, stainless steel cages within an environmentally controlled room.

## Safety evaluations

### Health observations

For all three studies, all dogs were observed twice daily, once in the morning and once in the afternoon, for mortality and clinical signs.

In the adult and juvenile studies, clinical examinations were performed twice daily for at least 7 days during acclimation (at least 4 h apart), at the time of dosing (on dosing days), continuously for 4 h post-dosing, and twice daily (at least 4 h apart) on non-dosing days. Veterinary physical examinations were performed once prior to randomization, once between randomization and dosing, every other week during the study period including 3 days before each dose cycle began, and on the day of the scheduled necropsy. The examination included evaluation of body temperature, body condition, capillary refill, ears, feces, urine, the oral cavity, and observations of general health and behavior, in addition to examination of the ocular (using an ophthalmoscope), integumentary, musculoskeletal (gait), nervous, gastrointestinal, lymphatic, cardiovascular (with stethoscope), and respiratory (with stethoscope) systems.

In the acute study, clinical examinations were performed daily for at least 7 days during acclimation, at the time of dosing, continuously for 8 h post-dosing, and at 12 and 36 h post-dosing. Veterinary physical observations were performed once prior to dosing and at approximately 4, 8, 24, and 48 h post-dosing. The examination included evaluation of body temperature, behavior, body condition, skin, eyes (with direct ophthalmoscope), capillary refill, oral cavity, ears, cardiovascular system (with stethoscope), respiratory system, gastrointestinal system, lymphatic system, musculoskeletal system/gait, neurologic system, feces and urine.

### Body weights

In the adult and juvenile studies, individual body weights were recorded 1 week prior to randomization, on the day prior to randomization, on study day −1, weekly during the study period including the day before each dose cycle, and on the day prior to the first day of the scheduled necropsy (nonfasted). Mean body weights were calculated for the corresponding intervals. Final body weights (fasted) were recorded on the day of the scheduled necropsy.

In the acute study, individual body weights were recorded 1 week prior to randomization, on the day of randomization, and on study day −1.

### Food consumption

Individual food weights were recorded daily during acclimation, beginning at least 7 days prior to randomization, and daily throughout the study period. Food consumption was calculated as g/dog/day. In the adult and juvenile studies, the weekly averages were reported for the corresponding body weight intervals.

### Clinical pathology

In the adult and juvenile studies, blood and urine samples for clinical pathology evaluations (hematology, coagulation, serum chemistry, and urinalysis) and fecal samples were collected from all dogs during acclimation prior to randomization (study week −1), on study day 80 (study week 11), and on the day of the scheduled necropsy. In the juvenile study, samples were collected at an additional time-point on study day 164 (study week 23). In the acute study, clinical pathology evaluation samples were only collected once during acclimation prior to randomization. Dogs were fasted overnight prior to blood collection while using cage pans for urine and fecal collection.

In all three studies, hematology profile included counts for erythrocytes, total leukocytes, differential counts for leukocytes (percent and absolute), platelets, and reticulocytes (percent and absolute); hemoglobin, hematocrit, mean corpuscular volume, mean corpuscular hemoglobin, mean corpuscular hemoglobin concentration, mean platelet volume, prothrombin time, activated partial thromboplastin time, fibrinogen, red cell distribution width, hemoglobin distribution width, platelet estimate, and red cell morphology.

In all three studies, serum chemistry profile included total protein, albumin, globulin, albumin/globulin ratio, total bilirubin, urea nitrogen, creatinine, alkaline phosphatase, alanine aminotransferase, aspartate aminotransferase, gamma glutamyl transferase, glucose, total cholesterol, calcium, chloride, potassium sodium, sorbitol dehydrogenase, triglycerides, creatine kinase, amylase, bicarbonate, lactate dehydrogenase, magnesium, total bile acid, and appearance (for degree of hemolysis, lipemia, and icterus). In the adult and juvenile studies, approximately 50 µL of serum was taken from each serum sample and shipped to AniLytics for determination of C-reactive protein levels.

In all three studies, urinalysis profile included specific gravity, pH, urobilinogen, total volume, color, clarity, protein, glucose, osmolality, bilirubin, occult blood, leukocytes, and microscopy of sediment. Fecal samples were examined for parasites and occult blood. Any abnormalities in color or consistency were also noted.

## Bioanalytical and exposure verification

For the adult and juvenile studies, blood samples for exposure verification were collected via the jugular vein from all dogs at approximately 24, 168, 336, and 504 h after the last dose of each dose cycle. The plasma samples were analyzed for the concentration of lotilaner, milbemycin A_3_ 5-oxime, and milbemycin A_4_ 5-oxime using a liquid chromatography tandem mass spectrometry method at WuXi AppTec.

Blood samples for bioanalytical and exposure verification were not collected for the acute study.

## Macroscopic and histology/microscopic evaluations

At the end of the adult and juvenile studies, dogs were humanely euthanized by an intravenous injection of sodium pentobarbital and exsanguinated. In the adult study, all dogs were euthanized on study day 197 or 198, 25 or 26 days following the last dose cycle. In the juvenile study, all dogs were euthanized on study day 256, 32 days following the last dose cycle. Death was confirmed by stethoscope. Complete and detailed gross and microscopic examinations on collected tissues were carried out on all dogs according to VICH GL 43 [12] under the supervision of veterinary pathologists.

In the acute study, all dogs survived to the completion of the study. As this study was designed to evaluate acute clinical signs, which would be expected to occur in the 48 h following accidental overdose, no necropsy or histopathology was performed, and the dogs were transferred to the CRL-Ashland colony. The use of the dogs in this manner respected the 3Rs of research (Replacement, Reduction and Refinement) as the two long-term studies evaluated the effects of repeated overdosing and histopathological changes were evaluated in these studies.

## Statistical methods

For the juvenile and adult studies, statistical analyses were conducted by BioSTAT Consultants according to VICH GL 43 [12]. Data were analyzed with the statistical software package SAS^®^ (version 9.2, SAS Institute Inc., Cary, NC, USA). Endpoints that were measured multiple times post-treatment and included a pre-treatment measurement (body weights, serum chemistry, coagulation, hematology, urinalysis, and body weight) were evaluated using repeated-measures linear mixed models (RMANCOVA) with “treatment,” “time,” and “sex” and associated two- and three-way interactions; and a covariate all as fixed effects. The pre-treatment value closest to dosing was used as the covariate. Endpoints measured once post-treatment that did not include a pre-treatment measurement (organ weights and organ weight ratios) were analyzed using analysis of variance (ANOVA) with “treatment,” “sex,” and “treatment by sex” as fixed effects. Depending on the significance of the interaction terms, treated groups were compared to control either within each sex (treatment-by-sex significant), within each time point (treatment-by-time significant) or main effect only (neither interaction significant but treatment main effect significant). All main effect, interaction terms, and pairwise comparisons of test article groups with the control were conducted at the 0.10 significance level, with the exception of the evaluation of treatment-sex-time which was conducted at the 0.05 significance level. In addition, all animal data were summarized through descriptive statistics or frequency counts. Summary statistics for the change from baseline (pre-treatment) body weights and clinical pathology variables were also calculated for each treatment group.

The acute study was a short-duration study; therefore, no inferential statistical testing was conducted.

## Results and discussion

### Dose administered

Dogs in all three studies were dosed in the fed state. As dogs were dosed using the various fixed tablet strengths based on their most recent body weights, doses were administered using the upper half of the nominal dose band. Target dosage levels in the adult and juvenile studies were 1.125 to 1.5 + 30 to 40, 3.375 to 4.5 + 90 to 120, and 5.625 to 7.5 + 150 to 200 mg/kg of MO + lotilaner, to the 1X, 3X and 5X groups, respectively. Individual dog doses were allowed to go over the targeted maximum, but not under the targeted minimum (i.e. a 1X dog could receive > 1.5 + 40 but not < 1.125 + 30 mg/kg of MO and lotilaner, respectively). Actual mean doses received across all dose cycles for the adult study were 1.34 + 35.7, 4.06 + 108.2, and 6.76 + 180.3 mg/kg MO + lotilaner. Actual mean doses received across all dose cycles for the juvenile study were 1.36 + 36.3, 4.10 + 109.2, and 6.75 + 179.9 mg/kg MO + lotilaner. The mean monthly doses can be found in Tables 2 and 3. Target dosage levels in the acute study were 1.5 + 40, 4.5 + 120, and 9.0 + 240 mg/kg of MO + lotilaner, to the 1X, 3X and 6X groups, respectively. Actual mean doses received for the acute study were 1.52 + 40.5, 4.50 + 119.9, and 9.03 + 240.9 mg/kg MO + lotilaner.

**Table 2:** Mean monthly milbemycin oxime dose (mg/kg) received in the adult and juvenile target animal safety studies

Study	Dose level	Mean milbemycin oxime dose (mg/kg)								
		Dose cycle 1	Dose cycle 2	Dose cycle 3	Dose cycle 4	Dose cycle 5	Dose cycle 6	Dose cycle 7	Dose cycle 8	Dose cycle 9
Adult	1X	1.35	1.27	1.39	1.34	1.32	1.36	1.34	N/A	N/A
	3X	4.11	4.03	3.99	4.02	4.15	4.04	4.07	N/A	N/A
	5X	6.74	6.67	6.91	6.74	6.82	6.73	6.73	N/A	N/A
Juvenile	1X	1.67	1.32	1.32	1.29	1.34	1.31	1.32	1.38	1.30
	3X	4.46	4.15	4.12	3.80	3.94	4.21	3.95	4.16	4.07
	5X	7.48	6.66	6.76	6.71	6.76	6.68	6.49	6.60	6.60

N/A: Not applicable

**Table 3:** Mean monthly lotilaner dose (mg/kg) received in the adult and juvenile target animal safety studies

Study	Dose level	Mean lotilaner dose (mg/kg)								
		Dose cycle 1	Dose cycle 2	Dose cycle 3	Dose cycle 4	Dose cycle 5	Dose cycle 6	Dose cycle 7	Dose cycle 8	Dose cycle 9
Adult	1X	35.9	33.9	36.9	35.7	35.1	36.3	35.8	N/A	N/A
	3X	109.7	107.5	106.4	107.3	110.5	107.7	108.4	N/A	N/A
	5X	179.7	177.8	184.1	179.7	181.9	179.5	179.4	N/A	N/A
Juvenile	1X	44.5	35.2	35.1	34.5	35.8	34.8	35.1	36.7	34.6
	3X	118.9	110.6	109.9	101.4	104.9	112.1	105.4	110.9	108.5
	5X	199.5	177.6	180.1	178.8	180.1	178.0	172.9	176.0	176.0

N/A: Not applicable

### Health observations

In all three studies, there were no treatment-related clinical or veterinary physical observations. All observations in the treated groups were noted with similar incidence in the control group, were limited to single dogs, were not noted in a dose-related manner, and/or were common findings for laboratory dogs. Clinical observations noted in these studies included blood loss, diarrhea, emesis, gingival erythema, hematochezia, increased salivation, injected sclera, lacrimation, reduced social behavior, loose stool, loss of condition, mucoid stool, mucus in eye, pinnal erythema, red face, retching, swollen vulva, vaginal discharge, and vaginal hemorrhage.

### Food consumption and body weights

In all three studies, food consumption was not statistically evaluated but values were generally similar across all groups throughout the study.

In the adult and juvenile studies, body weights were unaffected by treatment administration. In the adult study, mean pooled body weight in the 3X group was statistically significantly higher than the control group on study day 55 and mean pooled body weights in the 1X group were statistically significantly lower than the control group on study days 153 and 160. These results were transient and did not occur in a dose-related manner and were therefore not considered treatment-related. In the juvenile study, there were no statistically significant differences in body weight between the control and treated groups.

Due to the short duration of study (48 h), body weight changes were not measured during the acute study.

### Clinical pathology

In the adult and juvenile studies, there were no treatment-related effects on hematology, coagulation, serum chemistry, and urinalysis parameters. Statistically significant differences were noted but were not considered clinically relevant as there was no dose response, a lack of uniformity between sexes showed inconsistency of change (direction and/or magnitude) in treatment groups, had outlier values in some dogs skewing the assessments, and/or values were within CRL-Ashland historical control ranges. In addition, there were no clinically relevant treatment-related fecal findings.

In the acute study, clinical pathology was not evaluated post-dosing due to the short study duration.

### Macroscopic and microscopic examinations and organ weights

In the adult and juvenile studies, there were no treatment-related macroscopic, organ weight or microscopic observations in collected tissues. Any statistically significant differences in organ weights between any of the treated groups, relative to controls, were not considered toxicologically meaningful because there were no microscopic correlates to the weight changes, no dose–response relationships, organ weights were similar when adjusted for body weights or brain weights, opposite effects were present in males and females and/or the weights were within the CRL-Ashland historical control range. Any microscopic/histologic changes were considered incidental findings or related to some aspect of experimental procedures other than treatment administration. There was no treatment-related alteration in the prevalence, severity, or histologic character of those incidental tissue alterations. Some microscopic findings commonly found in the control group Beagles were noted sporadically in treated dogs and were not considered to be related to treatment administration due to lack of dose–response, presence of the finding in the control group(s), and/or presence of the finding in the CRL-Ashland historical control database. Microscopic findings in the reproductive tissues within the juvenile study were associated with the maturation and growth of the dogs during the study and not treatment related.

There are no macroscopic, organ weights, or microscopic results for the acute study as dogs were returned to colony at the end of the study.

### Exposure

Blood samples were collected in the long-term (juvenile and adult) studies to verify exposure, ensure the duration of the study was sufficient to cover the period of accumulation and verify safety at steady state plasma concentrations. Blood samples were not collected in the acute study as it was a single dose administration.

**Fig. 1:**
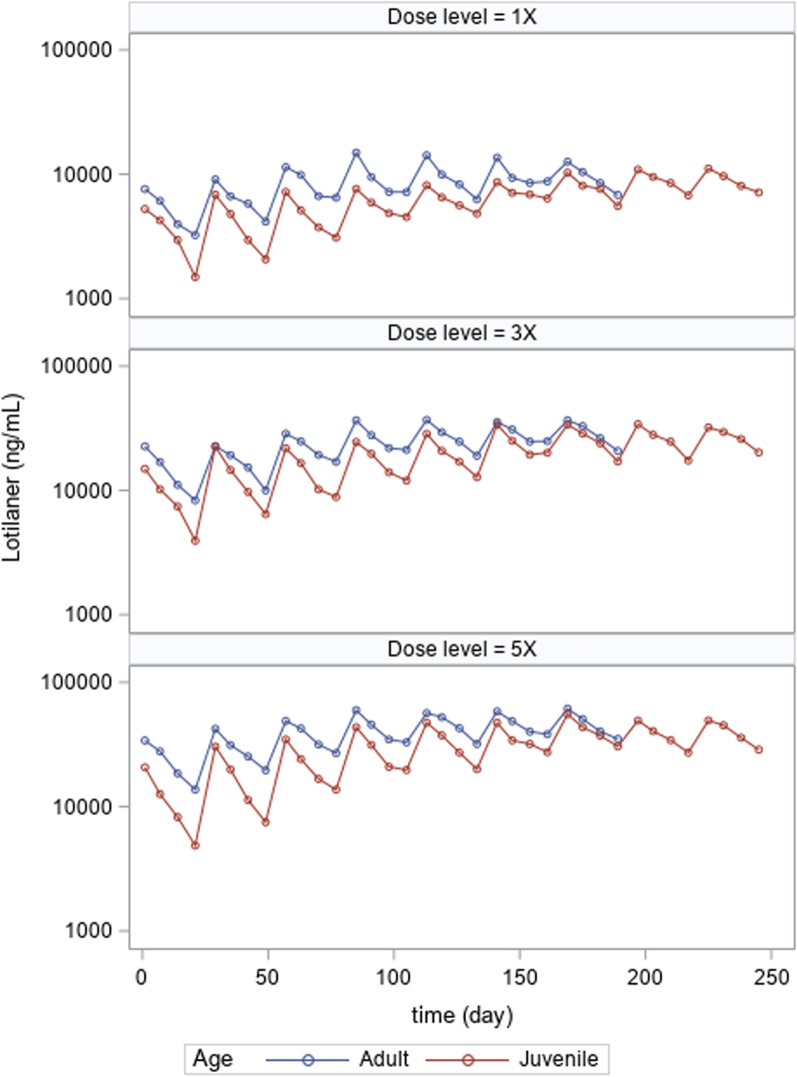
Mean lotilaner plasma concentrations in the adult and juvenile target animal safety studies following 1, 3 or 5X the recommended dose for seven and nine dose cycles, respectively

**Fig. 2:**
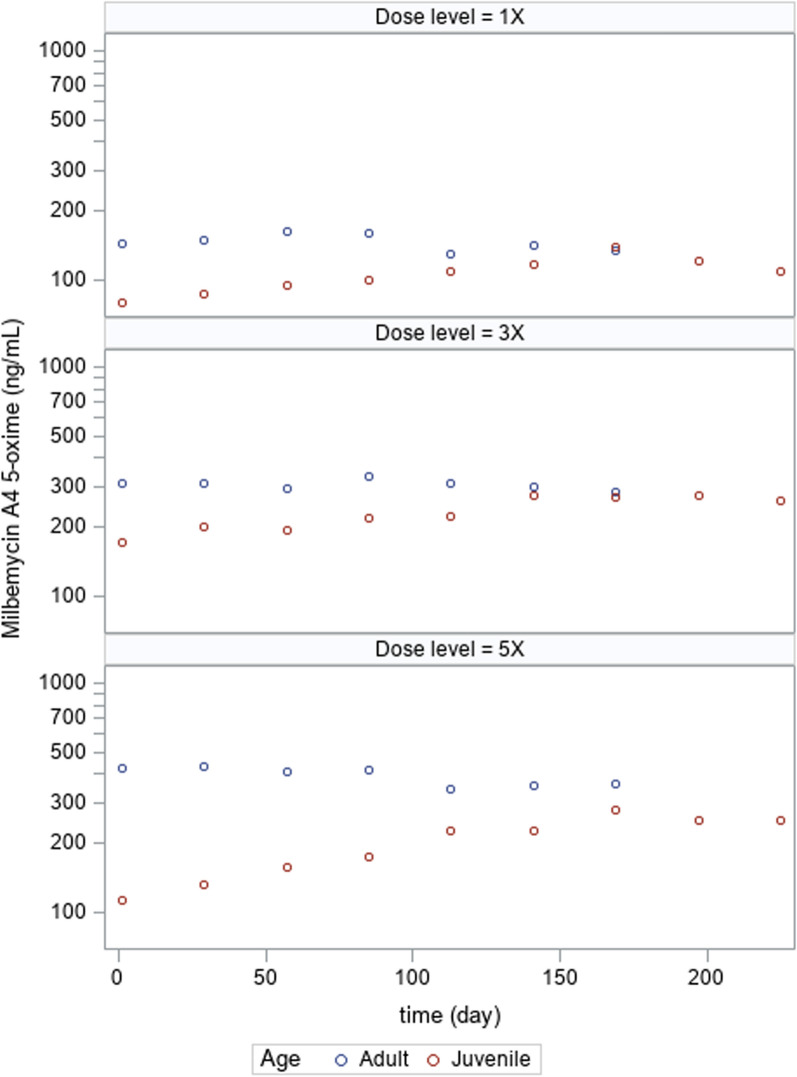
Mean milbemycin A_4_ 5-oxime plasma concentrations at 24 h post-dosing in the adult and juvenile target animal safety studies following 1, 3 or 5X the recommended dose for seven and nine dose cycles, respectively

In the adult and juvenile studies, following oral (tablet) administration of lotilaner and MO at clinical multipliers of 1X, 3X, and 5X, all dogs were exposed to lotilaner and MO. Exposure was similar in male and female dogs. Due to the blood sample collection scheme, whereas adequate profiling could be obtained for lotilaner (reported half-life ~ 30 days, [16, 18]), the shorter half-lives of MO A_3_ and A_4_ (reported half-lives of 33.9 and 77.2 h, respectively [19, 20]), resulted in limited plasma concentrations being measured at later time points. For this reason, Fig. 1 displays the full data collected for lotilaner, whereas in Fig. 2, only the 24-h time point for milbemycin A_4_ 5-oxime (the major factor comprising of approximately 80% of MO) is presented as all dogs had measurable concentrations for this time point (note: milbemycin A_3_ 5-oxime, the minor factor comprising approximately 20% of MO, is not presented but followed the same trends as A_4_). As measured by concentrations achieved at 24 h after each dose cycle, exposure for both lotilaner and MO increased as the clinical multiplier increased. The dose regimen used to achieve this multiplier (i.e., repeated daily dosing vs. single daily dose of 3 and 5X the amount) served to maximize total exposure rather than maximum concentration.

### Accumulation

In the adult study, accumulation based on concentrations achieved at 24 h after each dose cycle suggested that steady state lotilaner concentrations were < 2 times initial concentrations, were achieved by the fourth dose (day 88) and remained consistent for 4 months. There was no accumulation observed for either milbemycin A_3_ or A_4_ 5-oxime.

In the juvenile study, accumulation based on concentrations achieved at 24 h after each dose cycle suggested that steady state lotilaner and MO concentrations were two- to threefold higher than initial concentrations, were achieved by the sixth or seventh dose (day 140 or 168), and remained consistent for the remainder of the study.

Lotilaner, similar to other isoxazolines, has a long half-life and the resultant accumulation is expected [21]. This assures sustained efficacy over the entire month following treatment. At the same time, the accumulation of lotilaner did not result in increased incidence of adverse events. The difference between the time to steady state in juveniles and adults is likely related to changing physiology (i.e. blood volume, fat distribution, maturity of CYP enzymes, etc.) in the juveniles.

The lack of MO accumulation in the adult study is consistent with the shorter half-life of MO. As noted above, the perceived MO accumulation in juveniles could be related to changing physiology and not true accumulation as the MO plasma concentrations are more consistent with the adult concentrations as the juveniles aged. In fact, as can be seen in Figs. 1 and 2, for both lotilaner and MO, the plasma concentrations in the juveniles became more similar to the adult concentrations as the pups matured and achieved similarity around dose cycle 6 or 7 (day 140 or 168). Since the effective dose was confirmed in studies that were conducted in juveniles, there is no impact to efficacy from the lower blood concentrations observed in the juveniles.

## Conclusions

These three studies demonstrated that Credelio^®^ Plus has a wide safety margin when administered at monthly intervals to puppies and dogs at the high end of the commercial dose band. Careful clinical examinations, clinical pathology assessments and macroscopic/microscopic examinations in the long-term adult and juvenile safety investigations found that MO and lotilaner when co-administered as an oral chewable tablet formulation at up to 5X the upper end of the therapeutic dose range over at least 7 months to adult and juvenile dogs is well tolerated with no treatment-related effects. An additional safety evaluation at up to 6X the upper end of the therapeutic dose range to adult dogs as a single bolus dose was also well tolerated with no treatment-related effects.

As would be expected in Beagles [22], no signs of avermectin sensitivity were observed in the studies reported here. However, the safety of MO in dogs carrying the MDR 1 (ABCB1) mutation should be considered as these dogs are known to be more sensitive to macrocyclic lactones, including MO [23, 24]. The safety of MO in this sub-population of dogs has been extensively studied [25, 26] where doses between 5 and 10 mg/kg have shown mild and transient signs of avermectin sensitivity. In another study [27], MO was reported as safe at doses up to 10 mg/kg. Safety at a 5 mg/kg dose of MO provides reassurance of at least a 3X margin of safety of Credelio^®^ Plus in dogs carrying the MDR 1 (ABCB1) mutation.

The use of Credelio^®^ Plus as a broad spectrum endectocide that pet owners and veterinarians can use to effectively treat dogs with adult and immature intestinal nematode infections supports the recommendations from scientific expert groups such as ESCCAP, CAPC and TroCCAP to provide regular treatment and control of all intestinal nematodes of dogs and cats [28–30]. Furthermore, it is recommended by global veterinary practice guidelines (e.g. ESCCAP) that dogs exposed to flea and tick infestations in endemic regions of Europe due to their adverse clinical effects and the potential for disease transmission should be prescribed approved pulicidal and acaricidal products to provide consistent and ongoing efficacious flea and tick treatments [31, 32]. In total, these three safety studies demonstrated that Credelio^®^ Plus may be safely administered to both puppies and adult dogs each month in accordance with approved label and these recommendations.

## Data Availability

Datasets generated and/or analyzed during the current study are not publicly available due to commercial confidentiality of the research. Data not included in the manuscript can only be made available to bona fide researchers subject to a fully executed non-disclosure agreement.

## Abbreviations

ANOVA: Analysis of variance
ARRIVE: Animal Research: Reporting In Vitro Experiments
CAPC: Companion Animal Parasite Council
CFR: Code of Federal Regulations
CRL-Ashland: Charles River Laboratories, Ashland
ESCCAP: European Scientific Counsel Companion Animal Parasites
FDA: (United States) Food and Drug Administration
GLP: Good Laboratory Practice
LLC: Limited liability corporation
ML: Macrocytic lactone
MO: Milbemycin oxime
OECD: Organisation for Economic Co-operation and Development
RMANCOVA: Repeated-measures analysis of co-variance
SAS: Statistical Analysis System
SOPs: Standard operating procedures
TroCCAP: Tropical Council for Companion Animal Parasites Ltd.
VeDDRA: Veterinary Dictionary for Drug Regulatory Activities
VICH: Veterinary International Conference on Harmonization
